# Unconventional PINK1 localization to the outer membrane of depolarized mitochondria drives Parkin recruitment

**DOI:** 10.1242/jcs.161000

**Published:** 2015-03-01

**Authors:** Kei Okatsu, Mayumi Kimura, Toshihiko Oka, Keiji Tanaka, Noriyuki Matsuda

**Affiliations:** 1 Protein Metabolism Project, Tokyo Metropolitan Institute of Medical Science, Setagaya-ku, Tokyo 156-8506, Japan; 2 Laboratory of Protein Metabolism, Tokyo Metropolitan Institute of Medical Science, Setagaya-ku, Tokyo 156-8506, Japan; 3 Department of Life Science, College of Science, Rikkyo University, Nishi-Ikebukuro, Tokyo 171-8501, Japan

**Keywords:** Mitochondria, Parkin, Parkinson's disease, PINK1

## Abstract

Dysfunction of PTEN-induced putative kinase 1 (PINK1), a Ser/Thr kinase with an N-terminal mitochondrial-targeting sequence (MTS), causes familial recessive parkinsonism. Reduction of the mitochondrial membrane potential limits MTS-mediated matrix import and promotes PINK1 accumulation on the outer mitochondrial membrane (OMM) of depolarized mitochondria. PINK1 then undergoes autophosphorylation and phosphorylates ubiquitin and Parkin, a cytosolic ubiquitin ligase, for clearance of damaged mitochondria. The molecular basis for PINK1 localization on the OMM of depolarized mitochondria rather than release to the cytosol is poorly understood. Here, we disentangle the PINK1 localization mechanism using deletion mutants and a newly established constitutively active PINK1 mutant. Disruption of the MTS through N-terminal insertion of aspartic acid residues results in OMM localization of PINK1 in energized mitochondria. Unexpectedly, the MTS and putative transmembrane domain (TMD) are dispensable for OMM localization, whereas mitochondrial translocase Tom40 (also known as TOMM40) and an alternative mitochondrial localization signal that resides between the MTS and TMD are required. PINK1 utilizes a mitochondrial localization mechanism that is distinct from that of conventional MTS proteins and that presumably functions in conjunction with the Tom complex in OMM localization when the conventional N-terminal MTS is inhibited.

## INTRODUCTION

Parkinson's disease and its relative parkinsonism are pervasive neurodegenerative diseases. PTEN-induced putative kinase 1 (*PINK1*), a mitochondrial Ser/Thr kinase, was identified as a causal gene for familial recessive early-onset parkinsonism (Valente et al., 2004). Because patients with familial parkinsonism present symptoms similar to sporadic Parkinson's disease, functional analysis of familial Parkinson's disease-related proteins such as PINK1 provides insights into the pathogenic mechanism of the disease. Genetic studies using *Drosophila melanogaster* lacking *PINK1* showed a contribution of PINK1 to mitochondrial integrity *in vivo* (Clark et al., 2006; Park et al., 2006; Yang et al., 2006). *Drosophila* lacking *pink1* have abnormal mitochondrial morphology in flight muscles, short life span and male sterility (Clark et al., 2006; Park et al., 2006; Yang et al., 2006). These phenotypes are rescued by a component of the mitochondrial electron transport chain complex, a mitochondrial electron carrier or a positive regulator for mitochondrial protective genes (Koh et al., 2012; Vilain et al., 2012; Vos et al., 2012). Genes regulating mitochondrial morphology such as *Marf*, *opa1*, *fzo1* and *drp1* interact genetically with *PINK1* (Deng et al., 2008; Park et al., 2009; Poole et al., 2008; Yang et al., 2008). In addition, *Pink1*-knockout mice exhibit morphological and functional mitochondrial defects in striatum (Gautier et al., 2008; Kitada et al., 2007; Kitada et al., 2009). These reports suggest that *PINK1* plays important roles in maintaining mitochondrial robustness.

Recent cell-based and *in vivo* studies have revealed that PINK1 acts upstream of another gene product that is relevant to Parkinson's disease, Parkin (Clark et al., 2006; Geisler et al., 2010; Kitada et al., 1998; Matsuda et al., 2010; Narendra et al., 2010; Park et al., 2006; Rakovic et al., 2010; Vives-Bauza et al., 2010; Yang et al., 2006; Ziviani et al., 2010). PINK1 selectively recruits Parkin on depolarized mitochondria and phosphorylates both Parkin and ubiquitin, which leads to Parkin activation and the subsequent ubiquitylation of outer mitochondrial membrane (OMM) proteins on the damaged mitochondria (Chan et al., 2011; Iguchi et al., 2013; Kane et al., 2014; Kazlauskaite et al., 2014; Kondapalli et al., 2012; Koyano et al., 2014; Okatsu et al., 2012a; Sarraf et al., 2013; Shiba-Fukushima et al., 2012; Tanaka et al., 2010). Degradation of the ubiquitylated mitochondria is thought to proceed through the proteasome (Yoshii et al., 2011) and autophagy, a process referred to as mitophagy (Narendra et al., 2008; Okatsu et al., 2010).

During the aforementioned process, PINK1 recognizes a collapse of the membrane potential (ΔΨm) in mitochondria and signals this reduction to Parkin. In mitochondria with a normal ΔΨm, the positively charged mitochondrial-targeting sequence (MTS) of PINK1 is imported into the mitochondrial matrix and PINK1 undergoes stepwise cleavage; first by the mitochondrial processing peptidase (MPP), possibly with cooperation from ClpXP, and then intramembrane cleavage by presenilin-associated rhomboid-like protein (PARL) and possibly AFG3L2 (Deas et al., 2011; Greene et al., 2012; Jin et al., 2010; Meissner et al., 2011). Exposure of the phenylalanine (Phe) residue at position 104 of the N-terminus of processed PINK1 following PARL-mediated cleavage acts as a signal for ‘N-end rule pathway’-mediated degradation (Yamano and Youle, 2013). PINK1 is subsequently subjected to proteasomal degradation (Lin and Kang, 2008; Lin and Kang, 2010; Narendra et al., 2008) and the PINK1 signal is turned off under steady-state conditions. By contrast, dissipation of ΔΨm hinders movement of the positively charged MTS through the inner mitochondrial membrane (IMM), preventing exposure of the crucial Phe104 N-terminal processing site. PINK1 thus bypasses ΔΨm-dependent degradation, which triggers the accumulation of PINK1 on the OMM, interaction with the translocase of the outer membrane (TOM) complex, PINK1 dimerization and autophosphorylation (Lazarou et al., 2012; Matsuda et al., 2010; Narendra et al., 2010; Okatsu et al., 2012b). As a consequence, the PINK1 signal is turned on when ΔΨm decreases. A poorly understood aspect of this process is that when the ΔΨm-driven matrix targeting of MTS is inhibited, PINK1 is not released into the cytosol but is rather retained on the OMM. This contrasts with many matrix proteins that relocate to the cytosol following a decrease in ΔΨm. Consequently, the mechanism underlying PINK1 targeting to the OMM is crucial for PINK1 function. The molecular basis for PINK1 retention in the OMM of depolarized mitochondria and the domain(s) that are crucial to this process have not been conclusively resolved.

To date, various data on the mitochondrial localization signal and submitochondrial localization of PINK1 have been reported. For example, the submitochondrial localization of PINK1 varies from the OMM (Gandhi et al., 2006; Narendra et al., 2010; Weihofen et al., 2009; Zhou et al., 2008) to the intermembrane space (IMS) and IMM (Marongiu et al., 2009; Muqit et al., 2006; Silvestri et al., 2005). Moreover, there are conflicting conclusions regarding the extreme N-terminus of PINK1 (∼34 amino acids). This region has been reported to be sufficient for the mitochondrial localization of a reporter protein (Muqit et al., 2006; Silvestri et al., 2005; Takatori et al., 2008), but deemed dispensable for PINK1 mitochondrial localization (Zhou et al., 2008). To more fully understand the underlying processes, we must distinguish between the PINK1 mitochondrial localization mechanisms utilized for energized mitochondria and depolarized mitochondria.

Here, we dissect the multiple mitochondrial localization signals of PINK1 and disentangle the complicated mechanisms by using various deletion mutants and a newly established constitutively active mutant. We show that inactivation of the PINK1 N-terminus MTS alone is sufficient to promote PINK1 localization on the OMM through a second ‘latent’ outer mitochondrial membrane localization signal (OMS) that subsequently enhances the recruitment and activation of Parkin. Our results provide a molecular basis for how inhibition of PINK1 import through the IMM autonomously triggers PINK1 OMM localization, Parkin recruitment and mitochondrial degradation.

## RESULTS

### An N-terminal tag induces mitochondrial localization and autophosphorylation of PINK1

**Fig. 1.**
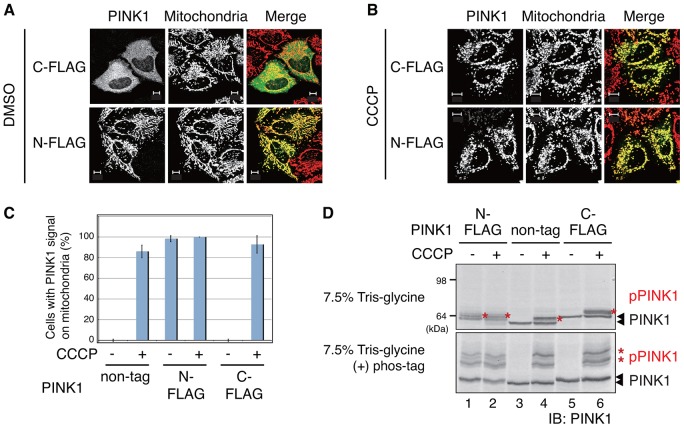
**Addition of an N-terminal FLAG tag causes PINK1 accumulation and activation on energized mitochondria.** (A,B) HeLa cells expressing N- or C-terminally FLAG-tagged PINK1 (N-FLAG–PINK1 or C-FLAG–PINK1) were immunostained using anti-PINK1 (green in merge) and anti-Tom20 (red in merge) antibodies in the absence (A) or presence (B) of CCCP treatment. N-FLAG–PINK1 localized to mitochondria even under steady-state conditions. Scale bars: 10 µm. (C) The number of cells with PINK1 localized to the mitochondria was counted in 100 cells. Data represent the mean±s.d. of at least three experiments. (D) The insertion of the N-FLAG tag converts PINK1 to its auto-phosphorylated form. Cells expressing non-tagged, N-FLAG-tagged or C-FLAG-tagged PINK1 were subjected to SDS-PAGE with (+) or without phos-tag and were immunoblotted (IB) for PINK1. The red asterisks indicate phosphorylated PINK1 (pPINK1).

Although PINK1 has been shown to localize on the OMM of depolarized mitochondria and undergo autophosphorylation-dependent activation in response to a decrease in ΔΨm, (Matsuda et al., 2010; Narendra et al., 2010; Okatsu et al., 2012b), the molecular basis of this process has yet to be elucidated. To begin to address this, we utilized a PINK1 construct with an epitope tag fused at the N-terminus that localizes on mitochondria in the absence of mitochondrial depolarization (Beilina et al., 2005; Takatori et al., 2008). HeLa cells were transfected with non-tagged PINK1 or PINK1 with an N-terminal FLAG tag (referred to as N-FLAG–PINK1) or a C-terminal tag (referred as C-FLAG–PINK1), and the localization of PINK1 was observed in the presence and absence of the mitochondrial uncoupler carbonyl cyanide *m*-chlorophenylhydrazine (CCCP). Consistent with previous reports using other N-terminal epitope tags such as Myc (Takatori et al., 2008), N-FLAG–PINK1 localized on mitochondria even without CCCP treatment (Fig. 1A), whereas C-terminally tagged and non-tagged PINK1 localized on mitochondria only following CCCP treatment (Fig. 1A–C) as reported previously (Matsuda et al., 2010). We next verified whether N-FLAG–PINK1 underwent phosphorylation using phosphorylated-protein-affinity SDS-PAGE (Phos-tag SDS-PAGE). The small molecule Phos-tag binds to acrylamide and two Mn^2+^ ions, such that phosphorylated proteins are captured by the Mn^2+^-Phos-tag and migrate more slowly than non-phosphorylated proteins (Kinoshita et al., 2006). Phos-tag SDS-PAGE showed that N-FLAG–PINK1 is constitutively phosphorylated, whereas C-FLAG–PINK1 and non-tagged PINK1 are phosphorylated only following CCCP-treatment (Fig. 1D).

### The PINK1 N-terminus contains a MTS

To address the molecular basis for N-FLAG–PINK1 accumulation on mitochondria, we first focused on the N-terminal region of PINK1, because the import of mitochondrial proteins frequently requires an intact MTS at the N-terminus. Both TargetP 1.1 (http://www.cbs.dtu.dk/services/TargetP/) and PSORT II (http://psort.hgc.jp/form2.html) predict mitochondrial localization for PINK1. The expected values for mitochondrial localization are 0.791 and 73.9% (the maximum values are 1 or 100%) in TargetP and PSORT II, respectively (supplementary material Fig. S1A). MTS functionality requires a positively charged amphiphilic α-helix within the N-terminus. Secondary structure prediction of the PINK1 N-terminus region utilizing Jpred3 (http://www.compbio.dundee.ac.uk/www-jpred/) and PHD (http://npsa-pbil.ibcp.fr/cgi-bin/npsa_automat.pl?page = /NPSA/npsa_phd.html) indicated the presence of a 20-amino-acid residue helical segment (supplementary material Fig. S1B). Visualization of this region as a helical wheel using the Phyre2 protein homology/analogy recognition engine (Phyre2: http://www.sbg.bio.ic.ac.uk/phyre2/html/page.cgi?id = index) suggested amphiphilicity (supplementary material Fig. S1C). In addition, the PINK1 N-terminal region is similar to the α-helix in the bacterial microcompartment shell protein PduB (4FAY) (supplementary material Fig. S1D,E), suggesting that the PINK1 N-terminus contains a genuine MTS.

**Fig. 2.**
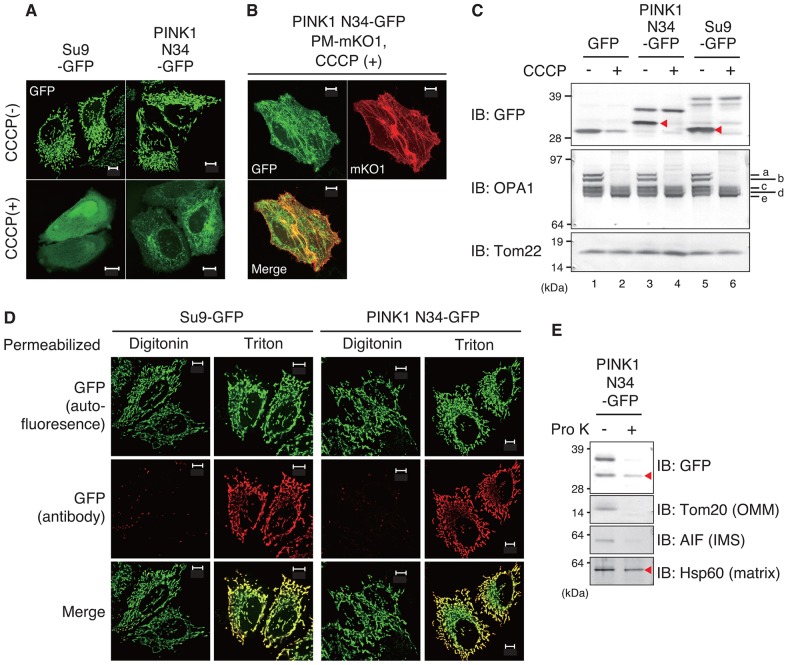
**The N-terminal 34 amino acids in PINK1 function as a mitochondrial-matrix-targeting signal that is dependent on ΔΨm.** (A) HeLa cells expressing PINK1-N34–GFP or Su9 (mitochondrial matrix marker) MTS–GFP with (+) or without (−) CCCP treatment. Cells were pretreated with CCCP for 2 h prior to transfection with Su9–GFP or PINK1-N34–GFP, and were further incubated in the presence of CCCP. (B) PINK1-N34–GFP partially colocalized with the plasma-membrane marker mKO1 (PM-mKO1) following CCCP treatment. (C) HeLa cells expressing GFP, PINK1-N34–GFP or Su9–GFP were treated with or without CCCP and then subjected to immunoblotting (IB). The red arrowheads indicate ΔΨm-dependent processed forms. OPA1 is detected as three bands following a decrease in ΔΨm. a–e indicate OPA1 mRNA spliced isoforms; a and b are processed in depolarized mitochondria. Tom22 represents the total amount of mitochondria. The size of GFP cleaved from N34–GFP appears to be greater than that of native GFP because a multi-cloning-site-derived peptide is added at the N-terminus of GFP. (D) HeLa cells expressing Su9–GFP or PINK1-N34–GFP were immunostained with an anti-GFP antibody after permeabilization with digitonin or Triton X-100, and then the immunofluorescence images were compared with the GFP autofluorescence images. Scale bars: 10 µm. (E) Mitochondria-rich fractions collected from HeLa cells expressing PINK1-N34–GFP were incubated with Proteinase K (Pro K: 250 µg/ml) and subjected to immunoblotting using anti-GFP, anti-Tom20 (OMM protein), anti-AIF (IMS protein) and anti-Hsp60 (matrix protein) antibodies. The red arrowheads indicate the Pro-K-resistant bands.

The 34 N-terminal amino acid residues (N34) of PINK1, which include the predicted amphiphilic α-helix, were fused with GFP and the subcellular localization of the resulting chimera (referred to as PINK1-N34–GFP) was examined. Consistent with previous reports (Muqit et al., 2006; Takatori et al., 2008), PINK1-N34–GFP localized to the mitochondria in HeLa cells, similar to Su9–GFP (Ishihara et al., 2003), an accepted mitochondrial matrix marker (Fig. 2A, upper panels). Incubation with CCCP inhibited the mitochondrial import of Su9–GFP, with fluorescence being limited to the cytosol and nucleus. The mitochondrial import of PINK1-N34–GFP was likewise inhibited by CCCP treatment; however, the reticular fluorescence pattern differed slightly from the cytosolic and nuclear pattern of Su9–GFP (Fig. 2A, lower panels). Because the processed form of PINK1-N34–GFP was almost undetectable following CCCP treatment (Fig. 2C), we believe that the unprocessed hydrophobic PINK1-N34–GFP localized in the membrane. Indeed, following CCCP treatment, the PINK1-N34–GFP fluorescence signal partially colocalized with the plasma-membrane-targeted mKO1 signal (Fig. 2B). To further confirm import into energized mitochondria, HeLa cells transfected with GFP, Su9–GFP and PINK1-N34–GFP were treated with CCCP and subjected to immunoblotting. Su9–GFP was cleaved in mitochondria under steady-state conditions, but remained intact when Su9–GFP import was inhibited by CCCP (Fig. 2C, lanes 5 and 6). PINK1-N34–GFP cleavage was similarly inhibited by blocking its import with CCCP (Fig. 2C, lanes 3 and 4). Five OPA1-immunoreactive bands were detected under steady-state conditions (Fig. 2C, odd lanes), whereas only three bands were detected following CCCP treatment (Fig. 2C, even lanes), confirming the dissipation of ΔΨm in this experiment (Ishihara et al., 2006).

To examine whether PINK1-N34–GFP localizes to the matrix, we immunostained HeLa cells with distinct permeabilization methods and performed a proteinase K (Pro K)-protection assay. Although Triton X-100 (1%) permeabilizes both OMM and IMM, digitonin (50 µg/ml) permeabilizes only the OMM. Tom20 (also known as TOMM20; an OMM protein) and cytochrome C (IMS protein) were clearly recognized by their respective antibodies after permeabilization with digitonin, and their signals overlapped well (supplementary material Fig. S2, left panel). Meanwhile, permeabilization with digitonin was insufficient to allow detection of the FoF1 ATP synthase β subunit (also known as ATP5B; exposed to matrix protein; Wang and Oster, 1998); instead, permeabilization with Triton X-100 was required (supplementary material Fig. S2, right panel). We then prepared HeLa cells expressing Su9–GFP and PINK1-N34–GFP, in which the GFP fluorescence was observed irrespective of the permeabilization conditions. Anti-GFP immunoreactivity was absent from both Su9–GFP- and PINK1-N34-GFP-expressing cells after permeabilization with digitonin, but became detectable following Triton X-100 permeabilization (Fig. 2D). We next prepared a mitochondria-enriched fraction from HeLa cells expressing PINK1-N34–GFP and incubated this with proteinase K (Pro K) before immunoblotting. PINK1–N34–GFP and the matrix protein Hsp60 are resistant to Pro K, whereas the OMM protein Tom20 and IMS protein AIF (also known as AIFM1) are susceptible to Pro K digestion (Fig. 2E). Using an *in vitro* import assay, Becker et al. also reported that the PINK1 N-terminus functions as a pre-sequence for mitochondrial import and that it passes through the IMM in a ΔΨm-dependent manner (Becker et al., 2012). Our data indicate that the N-terminal region of PINK1 functions as a MTS for mitochondrial matrix import.

### The import potential of PINK1 MTS is inhibited by the insertion of negatively charged amino acids

**Fig. 3.**
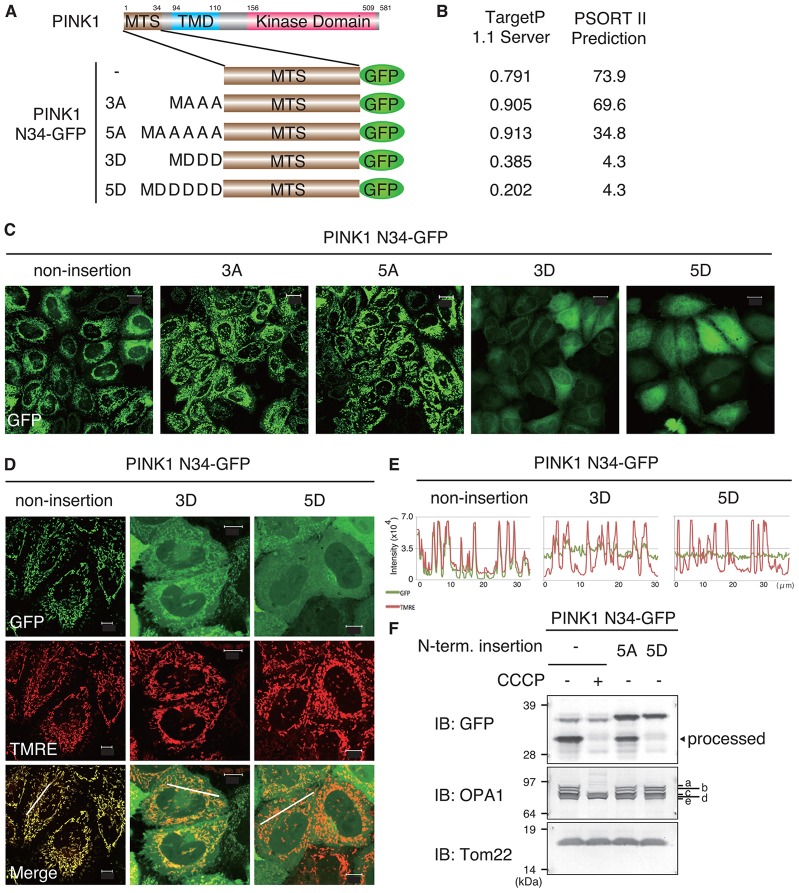
**The addition of negatively charged amino acids to the N-terminus of PINK1 MTS inhibits its mitochondrial import activity.** (A) Schematic diagram of the constructs used. (B) Mitochondrial localization prediction scores from TargetP 1.1 and PSORT II. (C) Autofluorescence images of HeLa cells expressing PINK1-N34–GFP with N-terminal insertion of the indicated amino acids. (D) HeLa cells expressing PINK1-N34–GFP mutants with 3 or 5 aspartic acid (‘D’) insertions were stained with the ΔΨm-dependent dye TMRE. Scale bars: 10 µm. (E) Graphs indicate the fluorescence intensity of the white lines in the merges in D. Green and red lines indicate the fluorescence intensity of GFP and TMRE (mitochondria), respectively. (F) The processed form of PINK1-N34–GFP that was detected by immunoblotting (IB) with an anti-GFP antibody was not detected in the 5D insertion. The OPA1 pattern indicates the presence of ΔΨm, and Tom22 represents total mitochondria as in Fig. 2C. a–e indicate OPA1 mRNA spliced isoforms; a and b are processed in depolarized mitochondria. The black arrowhead indicates the ΔΨm- and matrix-import-dependent processed form.

Because N-FLAG–PINK1 localized on mitochondria and underwent autophosphorylation (Fig. 1), we hypothesized that addition of extra amino acid residues at the N-terminus of PINK1 would impair the mitochondrial import potential of PINK1 N34. We tried to inactivate the PINK1 MTS sequence without disrupting the amphiphilic α-helix structure by inserting multiple Ala (non-polar) or Asp (negatively charged) amino acid residues into the PINK1 N-terminus (Fig. 3A). Mutants containing 3Ala, 5Ala, 3Asp and 5Asp yielded TargetP and PSORT II mitochondrial localization prediction scores of 0.905 and 69.6%, 0.913 and 34.8%, 0.385 and 4.3%, and 0.202 and 4.3%, respectively (Fig. 3B). We verified the predictions by observing the localization of these PINK1 mutants in HeLa cells. The Ala insertions had minimal effects on PINK1-N34–GFP localization, whereas the Asp insertions proportionally inhibited mitochondrial localization in relation to the number of residues inserted (Fig. 3C–E). To confirm these results, we examined the processing activity of the MTS by immunoblotting. The processed band was present in the 5Ala insertion mutant but absent from the 5Asp mutant (Fig. 3F), further indicating that Asp insertion almost completely inhibited mitochondrial import.

### Disruption of the N-terminal MTS induces an alternative signal-dependent mitochondrial localization mechanism and autophosphorylation of PINK1

**Fig. 4.**
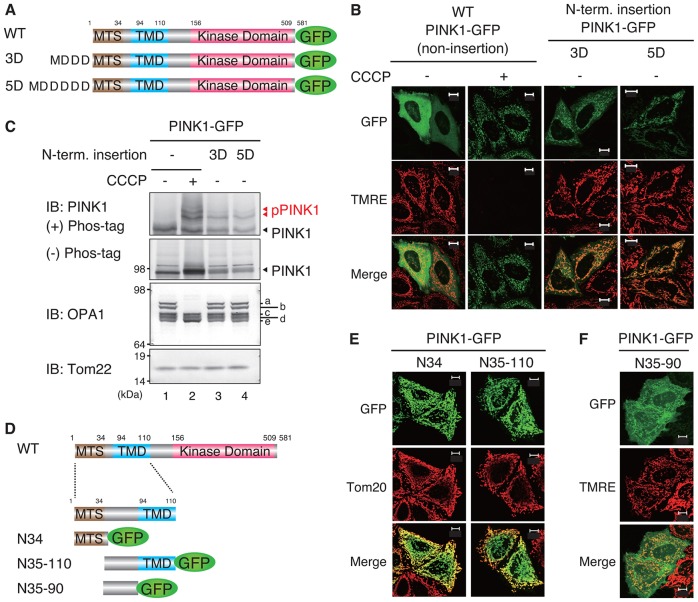
**Disruption of the N-terminal MTS induces alternative signal-dependent mitochondrial localization and autophosphorylation of PINK1.** (A) Schematic diagram of the constructs used in B and C. WT, wild type. (B) HeLa cells expressing full-length PINK1–GFP with the 3D or 5D insertions were imaged using GFP autofluorescence (green) and the ΔΨm-dependent dye TMRE (red). (C) The lysates were subjected to PAGE with (+) or without (−) PhosTag and immunoblotted (IB) with anti-PINK1, anti-OPA1 and anti-Tom22 antibodies. a–e indicate OPA1 mRNA spliced isoforms; a and b are processed in depolarized mitochondria. The red arrowheads indicate phosphorylated PINK1 (pPINK1). (D) Schematic diagram of the constructs used in E and F. (E) N35–110 of PINK1, which lacks the MTS, clearly targeted GFP to the mitochondria. (F) N35–90 of PINK1, which lacks both the MTS and TMD, still partially recruited GFP to the mitochondria. E and F reveal that the PINK1 N-terminus contains an alternative mitochondrial localization signal other than the MTS. Scale bars: 10 µm.

Because the Asp insertions inhibited PINK1-N34–GFP mitochondrial import, we examined the subcellular localization and phosphorylation of full-length PINK1–GFP when three or five Asp residues were inserted at the PINK1 N-terminus (Fig. 4A). The subcellular localization of PINK1–GFP, 3Asp-inserted PINK1–GFP or 5Asp-inserted PINK1–GFP was observed in relation to the ΔΨm-dependent dye TMRE. Under steady-state conditions, wild-type PINK1–GFP did not localize on energized mitochondria stained by TMRE, but did localize on TMRE-negative mitochondria following CCCP treatment (Okatsu et al., 2012b) (Fig. 4B, left two panels). Interestingly, insertions of 3Asp or 5Asp induced PINK1 accumulation on TMRE-positive polarized mitochondria (Fig. 4B, right two panels).

Because the full-length PINK1 that accumulates on depolarized mitochondria is phosphorylated (Okatsu et al., 2012b), we examined the phosphorylation of PINK1–GFP with Asp insertions using Phos-tag SDS-PAGE. Whereas wild-type PINK1–GFP undergoes phosphorylation only following CCCP treatment (Fig. 4C, lanes 1 and 2), PINK1–GFP with either of the Asp-repeat insertions was phosphorylated even under steady-state conditions (Fig. 4C, lanes 3 and 4). These results suggest that disruption of the PINK1 MTS by insertion of the negatively charged amino acids induced mitochondrial localization and autophosphorylation of full-length PINK1. Because the FLAG epitope tag contains five Asp residues (DYKDDDDK), it is reasonable to assume that placement of the FLAG tag at the N-terminus disrupts the PINK1 MTS and induces mitochondrial localization and autophosphorylation (Fig. 1).

Results shown in Fig. 4B,C contrast with the largely cytosolic localization data of PINK1-N34–GFP with the Asp insertions (Fig. 3C). However, given that PINK1 possesses an alternative OMM localization signal that functions when the N-terminal MTS is inhibited, this seemingly contradictory result is explainable. We sought to define the second PINK1 mitochondrial localization signal. As in the case of N-terminal MTS identification (supplementary material Fig. S1), we first tried *in silico* identification. A Kyte–Doolittle plot (Kyte and Doolittle, 1982) suggested that in addition to the MTS and putative transmembrane domain (TMD; residues 94–110), there is a weak hydrophobic region around residues 70–95 of the PINK1 N-terminus (supplementary material Fig. S3A). Jpred3, PHD and Phyre2 search engines also suggest a helical structure for this region (supplementary material Fig. S3B). Moreover, TargetP 1.1 and PSORT II estimate that residues 67–94 of PINK1, a region proximal to the TMD, has the potential to function as a mitochondrial localization domain (supplementary material Fig. S3C). We next constructed several fluorescent chimeras of GFP fused with various deletions of the 110 amino acids comprising the PINK1 N-terminus (Fig. 4D), and examined their subcellular localization. N35–110 of PINK1, which lacks a MTS, clearly targeted GFP to the mitochondria (Fig. 4E), as did N35–90 albeit with a weaker signal (Fig. 4F). These results reveal that, in addition to the typical N-terminal MTS, PINK1 contains a second mitochondrial localization signal spanning residues 35–110. This is consistent with a previous study reporting that PINK1 lacking the first 34 residues still localizes on the mitochondria (Zhou et al., 2008).

### Loss of the MTS in the N-terminus induces mitochondrial localization and autophosphorylation of PINK1

Because functional disruption of the PINK1 MTS (N34) causes mitochondrial localization and activation of PINK1 (Fig. 4), we next constructed PINK1 lacking the MTS (N34) and examined its localization and phosphorylation. We performed immunocytochemistry using an anti-PINK1 antibody (the antigenic region corresponds to amino acids 175–250), an anti-Tom20 antibody (mitochondrial marker), and MitoTracker Orange CM-H_2_TMROS (a fixable ΔΨm-sensitive dye). We confirmed that there was no crosstalk between the respective fluorescent signals (supplementary material Fig. S4A). Full-length PINK1 specifically localized on depolarized mitochondria as in Fig. 4B, whereas PINK1 lacking N34 (referred to as ΔN34 PINK1) localized on mitochondria with an intact ΔΨm as did N-FLAG–PINK1 (supplementary material Fig. S4B,C). We next analyzed the phosphorylation state of ΔN34 PINK1 using Phos-tag PAGE. Although the phosphorylation signal was weaker than that of N-FLAG–PINK1 (Fig. 1D), ΔN34 PINK1 was also phosphorylated in the absence of CCCP treatment (supplementary material Fig. S4D, lane 3). These results indicate that removal of the MTS promotes mitochondrial localization and autophosphorylation of full-length PINK1.

### Mitochondrial translocase Tom40 is important in mitochondrial localization and phosphorylation of constitutively active PINK1 mutants

**Fig. 5.**
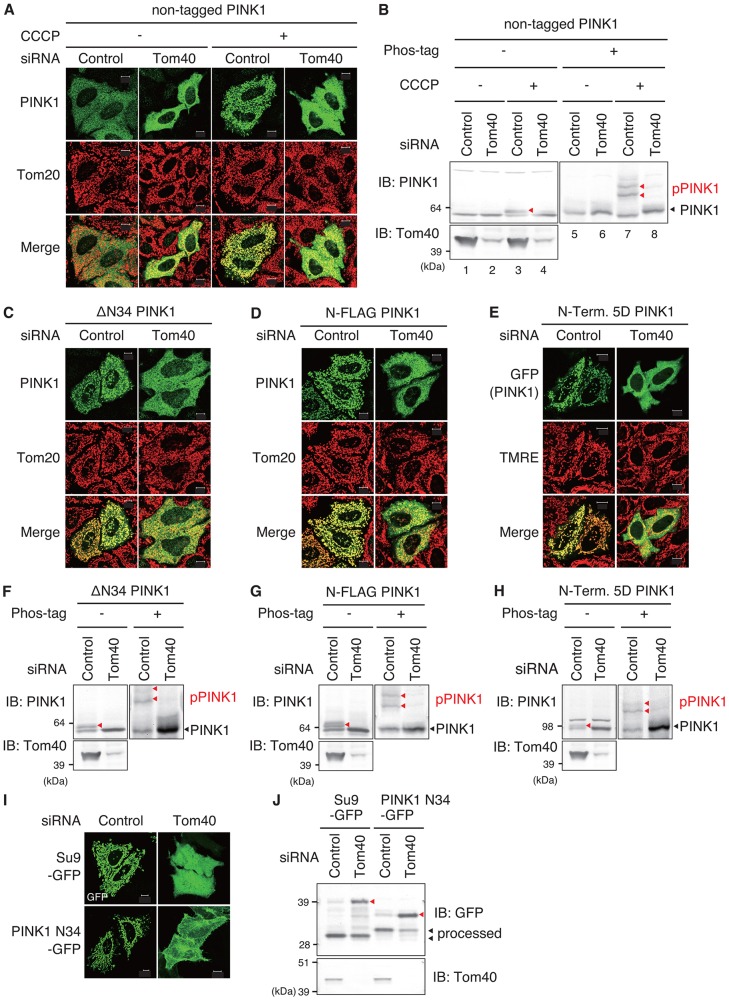
**Autophosphorylation and OMM localization of PINK1 depend on Tom40.** (A,B) HeLa cells pretreated with Tom40 siRNA were transfected with non-tagged PINK1, and then treated with or without CCCP (10 µM, 1 h). These cells were subjected to immunostaining with anti-PINK1 and anti-Tom20 antibodies (A), and lysates were immunoblotted (IB) with anti-PINK1 and anti-Tom40 antibodies after SDS-PAGE with (+) or without (−) Phos-tag (B). (C–H) HeLa cells treated with Tom40 siRNA were subsequently transfected with N-terminally deleted ΔN34 PINK1 (C,F), N-terminally FLAG-tagged PINK1 (D,G) or N-terminally 5D-inserted PINK1–GFP (E,H). These cells were fixed and immunostained with anti-PINK1 and anti-Tom20 antibodies (C,D) or were imaged using GFP autofluorescence and TMRE in living cells (E). The cell lysates were immunoblotted with anti-PINK1 and anti-Tom40 antibodies after SDS-PAGE with or without Phos-tag (F–H). The red and black arrowheads indicate phosphorylated full-length PINK1 (pPINK1) or non-phosphorylated full-length PINK1, respectively. (I,J) Mitochondrial localization (I) and MTS processing (J) of both the mitochondrial matrix markers Su9–GFP and PINK1-N34–GFP were inhibited by Tom40 knockdown, confirming that Tom40 knockdown indeed hampers matrix targeting of MTS. Scale bars: 10 µm.

In general, mitochondrial proteins containing MTS are imported through Tom40, a channel component of the translocase of the outer membrane (TOM) complex. Thus, we expected that Tom40 knockdown would inhibit MTS-dependent mitochondrial import, resulting in PINK1 constitutive mitochondrial localization, similar to MTS disruption (Fig. 4) or MTS deletion (supplementary material Fig. S4). To test this, HeLa cells were transfected with both Tom40 small interfering (si)RNA and non-tagged PINK1, and the localization and phosphorylation state of PINK1 was examined. In contrast to PINK1 MTS disruption (Fig. 4B) and MTS deletion (supplementary material Fig. S4B), PINK1 neither localized on energized mitochondria (Fig. 5A, left two panels) nor was phosphorylated (Fig. 5B, lane 6). Rather, the downregulation of Tom40 hampered PINK1 localization (Fig. 5A, right two panels) and phosphorylation (Fig. 5B, lane 8) on depolarized mitochondria. We also observed the effect of Tom40 knockdown on OMM localization and autophosphorylation of MTS-deficient PINK1 mutants. The mitochondrial localization of ΔN34 PINK1, N-FLAG–PINK1 and 5Asp PINK1 were blocked in cells treated with Tom40 siRNA (Fig. 5C–E), as was autophosphorylation (Fig. 5F–H). Import and MTS processing of Su9–GFP and PINK1-N34–GFP were also inhibited (Fig. 5I,J), confirming that knockdown of Tom40 indeed hampers matrix targeting of the MTS. Unlike PINK1 MTS dysfunction following insertion of 5Asp or MTS deletion (e.g. ΔN34), MTS dysfunction in response to Tom40 knockdown did not enhance PINK1 localization and activation in OMM. Tom40 is thus involved in OMM localization and autophosphorylation of PINK1, as suggested by previous reports (Hasson et al., 2013; Lazarou et al., 2012; Okatsu et al., 2013) (see Discussion).

### The second mitochondrial localization signal allows PINK1 to localize on OMM when the typical MTS is inhibited

**Fig. 6.**
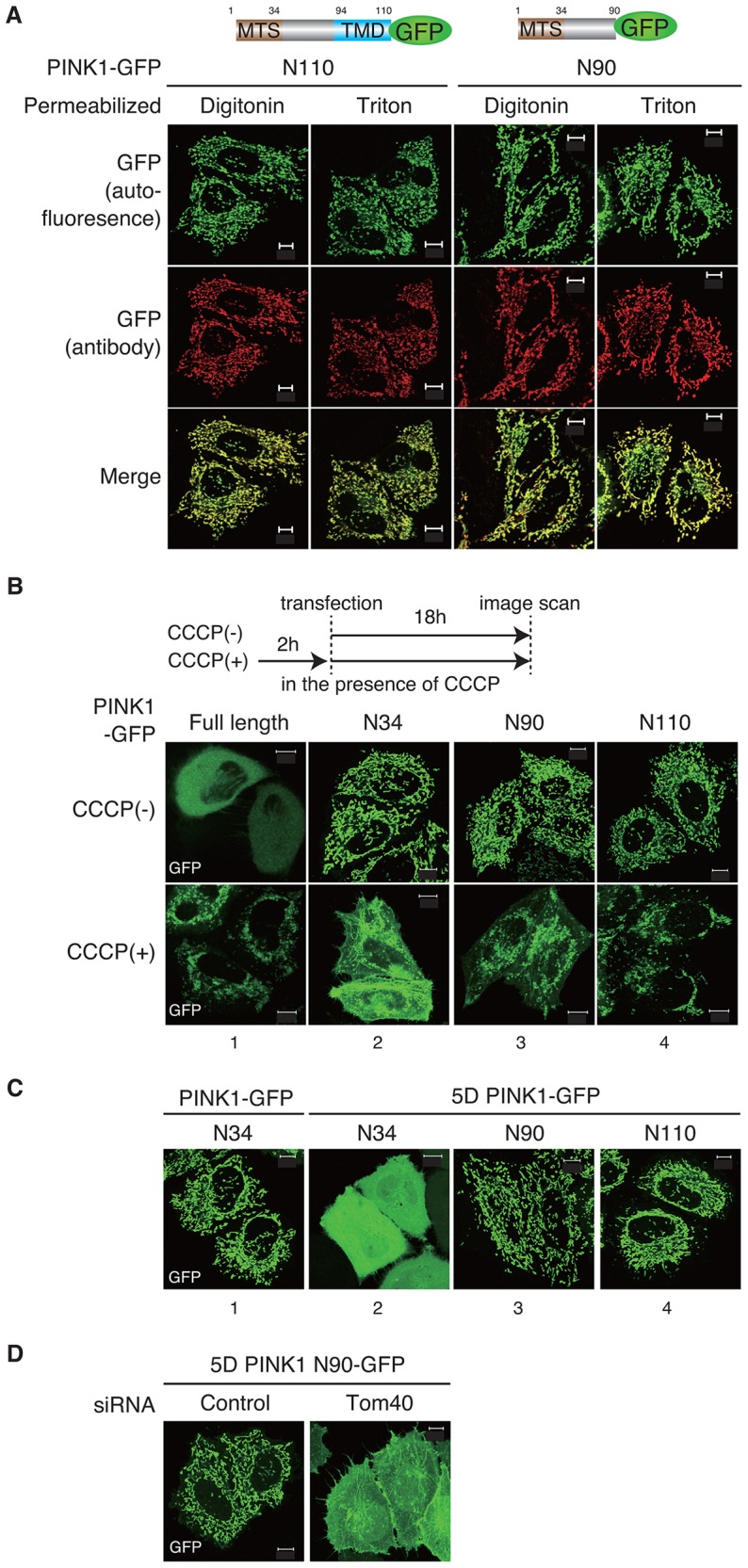
**The N-terminal 90 amino acids of PINK1 are sufficient for mitochondrial localization when typical MTS function is inhibited.** (A) GFP fused to the N-terminal 90 or 110 amino acids of PINK1 does not pass through the IMM. Permeabilization conditions did not affect the immunostaining pattern of either PINK1-N110–GFP or N90–GFP as detected by an anti-GFP antibody. (B) Mitochondrial localization of PINK1-N90–GFP and N110–GFP was conserved even following CCCP treatment. (C) Mitochondrial localization of PINK1-N90–GFP and N110–GFP was unchanged when five Asp residues were inserted at the N-terminus to inhibit MTS function. (D) Tom40 knockdown hampered the mitochondrial localization of 5D PINK1-N90–GFP, suggesting that Tom40 is required for function of the alternative mitochondrial localization signal. Scale bars: 10 µm.

We next examined whether the second mitochondrial localization signal is essential for OMM localization of PINK1. As shown in Fig. 2D, in the case of matrix-localized PINK1-N34–GFP, anti-GFP immunoreactivity was absent after digitonin permeabilization, but became detectable following Triton X-100 permeabilization. By contrast, in the case of PINK1-N90–GFP and PINK1-N110–GFP, their anti-GFP antibody immunostaining pattern was not affected by the permeabilization conditions (Fig. 6A). These results indicate that the domain downstream of the N-terminal MTS promoted PINK1 retention in the OMM (Fig. 6A), whereas PINK1 lacking this domain (such as PINK1-N34–GFP) passed through the IMM to the matrix in energized mitochondria (Fig. 2D). Because N110–GFP contains the TMD domain for PARL-mediated processing, it is possible that Phe104 is exposed by processing and N110–GFP is subjected to N-end-rule-catalyzed degradation. However, N110–GFP seemed not be degraded, probably because an unidentified domain just behind the TMD is essential for degradation or because the proximal GFP tag inhibits PARL-mediated processing owing to structural hindrance.

We then examined the subcellular localization of the chimera proteins under ΔΨm dissipation conditions. Although pre-treatment with CCCP caused PINK1-N34–GFP to localize to the cytosol and plasma membrane (Fig. 6B, panel 2, also shown in Fig. 2), mitochondrial localization was retained in the PINK1-N90–GFP and N110–GFP chimeras following CCCP treatment (Fig. 6B, panels 3 and 4). We also examined localization following the insertion of 5Asp at the N-terminus to inhibit MTS function (Fig. 3). As before, the insertion changed the mitochondrial localization of PINK1-N34–GFP to a dispersed cytosolic pattern (Fig. 6C, panel 2, also shown in Fig. 3), whereas the mitochondrial localization of PINK1-N90–GFP and N110–GFP remained unchanged (Fig. 6C, panels 3 and 4). These results suggest that the second mitochondrial localization signal residing between residues 35–90 maintains PINK1 localization in the OMM when the MTS is inhibited. By contrast, Tom40 knockdown hampered mitochondrial localization of 5D PINK1-N90–GFP, confirming that Tom40 is required for the function of the alternative OMM localization signal (Fig. 6D).

### The putative TMD is dispensable for OMM localization of PINK1 on depolarized mitochondria

**Fig. 7.**
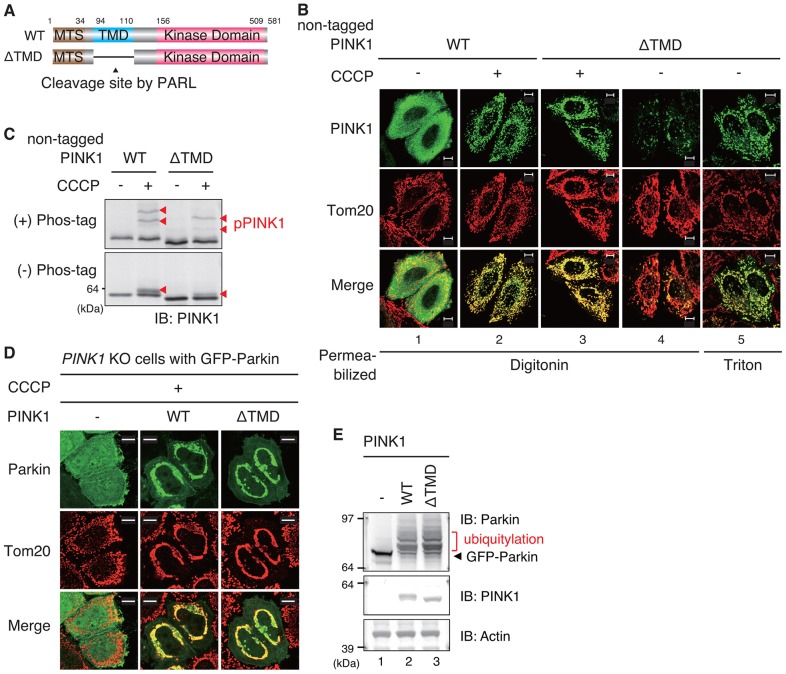
**The TMD of PINK1 is required for stop-transfer in the IMM in energized mitochondria but is dispensable for OMM localization in depolarized mitochondria.** (A) Schematic diagram of the constructs used in this figure. WT, wild-type. (B) Subcellular localization of non-tagged wild-type PINK1 or ΔTMD PINK1 (lacking the putative TMD, residues 94–110) under normal and ΔΨm dissipation conditions. The TMD functions as a stop-transfer signal to inhibit passage through the IMM in energized mitochondria, whereas the TMD is dispensable for localization in depolarized mitochondria. (C) ΔTMD PINK1 undergoes autophosphorylation following CCCP treatment. Red arrowheads indicate phosphorylated PINK1 (pPINK1). (D) *PINK1*-knockout cells coexpressing GFP–Parkin and the indicated PINK1 mutants were immunostained with anti-GFP and anti-Tom20 antibodies. Parkin was recruited to depolarized mitochondria by ΔTMD PINK1, confirming that ΔTMD PINK1 correctly localized on the OMM of depolarized mitochondria. Scale bars: 10 µm. (E) *PINK1*-knockout cell lysates coexpressing GFP–Parkin and wild-type or ΔTMD PINK1 were immunoblotted (IB) with anti-Parkin, anti-PINK1 and anti-actin (loading control) antibodies. The red bar indicates ubiquitylated GFP–Parkin. Although the position of the molecular-mass marker (64 kDa) changes a little between a hand-made non-phostag gel (C) and the commercial precast gel (E), PINK1 bands shown in E correspond to the full-length PINK1.

Next, we examined whether the putative TMD (residues 94–110) is essential for OMM localization following CCCP treatment, because this hydrophobic region was defined *a priori* as a PINK1 TMD (Silvestri et al., 2005). We expressed non-tagged wild-type PINK1 or PINK1 without the putative TMD (referred to as ΔTMD PINK1) in cells (Fig. 7A) and examined their subcellular localization. Under steady-state conditions in the absence of CCCP treatment, anti-PINK1 immunoreactivity of ΔTMD PINK1 was barely detectable after permeabilization with digitonin, but was readily apparent following Triton X-100 permeabilization (Fig. 7B, panels 4 and 5). These results revealed that the hydrophobic TMD domain (residues 94–111) of PINK1 functions as stop-transfer signal to inhibit passage through the IMM in energized mitochondria, as suggested previously (Becker et al., 2012), and that it supports IMM-based cleavage at Phe104 and subsequent PINK1 degradation (Yamano and Youle, 2013). We then examined whether this putative TMD is responsible for PINK1 localization in the OMM of depolarized mitochondria. Surprisingly, ΔTMD PINK1 still localized on mitochondria following CCCP treatment (Fig. 7B, panel 3). PINK1 ΔTMD received autophosphorylation following CCCP treatment (Fig. 7C), suggesting that this mutant PINK1 localizes correctly as does wild-type PINK1. Moreover, when ΔTMD PINK1 was coexpressed with GFP–Parkin in *PINK1*-knockout cells, ΔTMD PINK1 assisted the recruitment of GFP–Parkin to depolarized mitochondria and its activation, equivalent to wild-type PINK1 (Fig. 7D,E). These results further confirm that ΔTMD PINK1 correctly localizes on the OMM of depolarized mitochondria, and that TMD is dispensable for the OMM retention of PINK1 in depolarized mitochondria.

Data shown in Figs 2–7 suggest that PINK1 possesses an alternative mitochondrial localization signal between residues 34 and 90, and that this signal presumably interacts with the Tom complex in OMM localization when the primary N-terminal MTS is inhibited, i.e. following a decrease in ΔΨm (see Discussion).

### Inhibition or deletion of the PINK1 MTS drives Parkin mitochondrial localization and activation

**Fig. 8.**
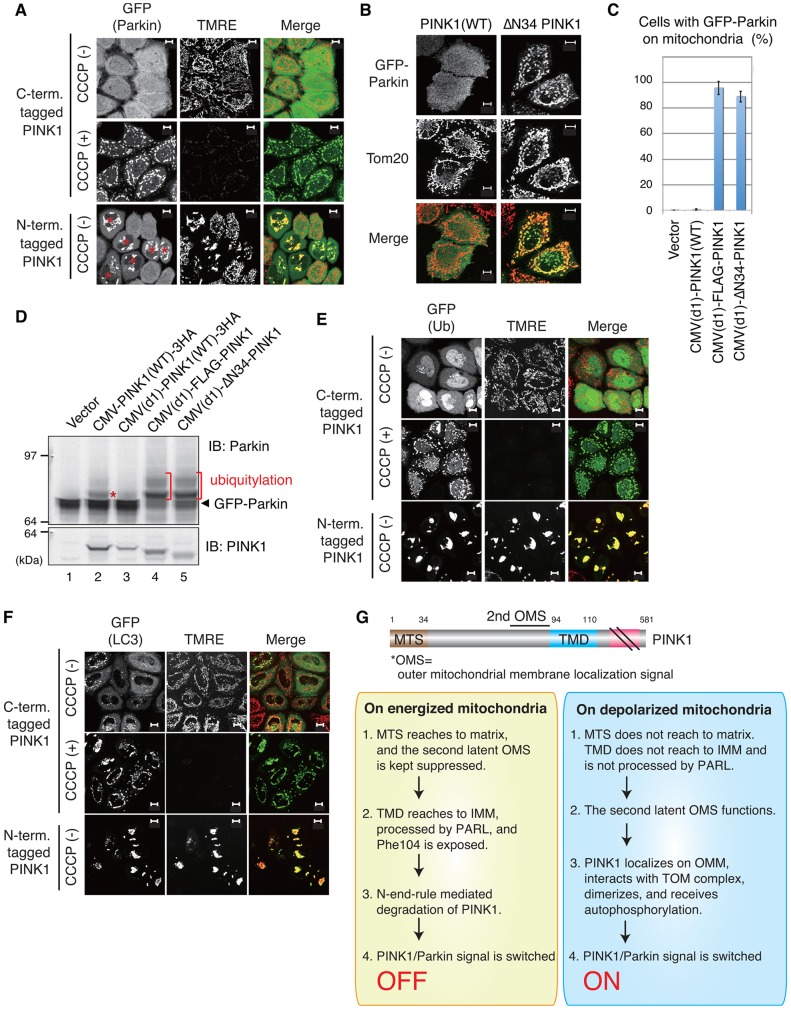
**N-FLAG–PINK1 and ΔN34 PINK1 activate and recruit Parkin to energized mitochondria followed by ubiquitin and LC3 accumulation.** (A) Living HeLa cells stably expressing GFP–Parkin are transfected with weak-promoter-driven N-terminally tagged or C-terminally tagged PINK1 and stained with the ΔΨm-dependent dye TMRE. The cells treated with CCCP are not stained with TMRE, and GFP–Parkin is localized on the depolarized mitochondria. By contrast, N-FLAG–PINK1 expression induces Parkin localization on TMRE-stained mitochondria. The red asterisks indicate cells transfected with N-FLAG–PINK1 that causes mitochondrial aggregation. (B) HeLa cells coexpressing GFP–Parkin and weak-promoter-driven wild-type (WT) or ΔN34 PINK1 were immunostained with anti-GFP and anti-Tom20 antibodies. (C) A graph showing the number of cells with Parkin localized to the mitochondria when GFP–Parkin and the indicated PINK1 mutants were coexpressed. Data represent the mean±s.d. of at least three experiments in 100 cells. (D) Lysates from HeLa cell coexpressing GFP–Parkin and the indicated PINK1 mutants were immunoblotted with anti-Parkin and anti-PINK1 antibodies. The red asterisk and bars indicate ubiquitylated GFP–Parkin. The PINK1 band shown in the lower panel corresponds to the full-length PINK1. (E,F) Ubiquitin (E) and LC-3 (F) accumulate on only depolarized mitochondria in C-terminally tagged PINK1-expressing cells. Meanwhile, N-FLAG–PINK1 triggered obvious accumulation of both ubiquitin (E) and LC-3 (F) on TMRE-stainable healthy mitochondria. Scale bars: 10 µm. (G) Our model for PINK1 localization on the OMM of depolarized mitochondria. See text for details.

Parkin recruitment and E3 activity on depolarized mitochondria completely depend on PINK1. Thus, we finally examined whether PINK1 that is localized on the OMM and autophosphorylated following MTS dysfunction (i.e. N-terminal tag or MTS deletion) recruits and activates Parkin on energized mitochondria. To confirm whether N-FLAG–PINK1 or ΔN34 PINK1 activates Parkin under steady-state conditions, we tried to examine the localization and E3 activity of Parkin when these PINK1 mutants were expressed. However, overexpression of exogenous PINK1 itself (even the wild-type form) triggers mitochondrial localization and autoubiquitylation of Parkin even in the absence of CCCP (Matsuda et al., 2010; Narendra et al., 2010), and thus comparisons of the effect that the PINK1 mutants have on Parkin are problematic. To overcome this limitation, we have previously established more appropriate experimental conditions for the coexpression of PINK1 and Parkin using a weakened CMV promoter – CMV(d1) (Okatsu et al., 2012b). Overexpression of wild-type PINK1 using an intact CMV promoter causes Parkin mitochondrial localization and activation irrespective of ΔΨm, whereas expression of wild-type PINK1 using the CMV(d1) promoter results in Parkin recruitment and activation that are dependent on a decrease in ΔΨm (Okatsu et al., 2012b). HeLa cells stably expressing GFP–Parkin were transfected with N-terminally or C-terminally tagged PINK1 under control of the CMV(d1) promoter, and then subjected to TMRE staining. In cells expressing C-terminally 3HA-tagged PINK1, Parkin was cytosolic under steady-state conditions and mitochondria were stained with TMRE. By contrast, CCCP treatment promoted Parkin localization on unstained mitochondria (Fig. 8A, upper and the middle panels). Interestingly, transfection of N-terminal FLAG-tagged PINK1 triggered Parkin recruitment on energized mitochondria stained with TMRE (Fig. 8A, lower panels). Similarly, ΔN34 PINK1 recruited GFP–Parkin to energized mitochondria even without CCCP treatment, whereas wild-type PINK1 did not recruit Parkin to mitochondria under the same experimental conditions (Fig. 8B). Statistical analysis using HeLa cells co-transfected with GFP–Parkin and N-FLAG–PINK1 or ΔN34 PINK1 confirmed their Parkin recruitment activity (Fig. 8C). We next examined whether the expression of N-FLAG–PINK1 and ΔN34 PINK1 accelerate the autoubiquitylation of GFP–Parkin, because the translocated Parkin exerts E3 activity and ubiquitylates in-frame-fused GFP, and thus autoubiquitylation can be used as an index of Parkin activation (Matsuda et al., 2010). N-FLAG–PINK1 and ΔN34 PINK1 under the CMV(d1) promoter accelerated autoubiquitylation of GFP–Parkin, whereas C-terminal 3×HA-tagged PINK1 under the same CMV(d1) promoter did not (Fig. 8D, lanes 3–5). Indeed, Parkin E3 activity was more pronounced with the N-terminal-tagged and N-terminal-deleted PINK1 than with over-produced wild-type PINK1 (Fig. 8D, compare lanes 2 with lanes 4 and 5), even though the PINK1 level of the two mutants was lower than that of wild-type PINK1 under the full CMV promoter (Fig. 8D, lower panel). We further examined whether N-FLAG–PINK1 triggers downstream events such as the recruitment of ubiquitin and LC-3, an autophagic marker (Kabeya et al., 2000) on mitochondria. As has already been reported, ubiquitin and LC-3 accumulated only following CCCP treatment under normal conditions, i.e. in C-terminal-tagged PINK1-expressing cells (Narendra et al., 2008; Okatsu et al., 2010). N-FLAG–PINK1 triggered obvious accumulation of both ubiquitin and LC-3 on TMRE-stainable healthy mitochondria (Fig. 8E,F). These results indicate that dysfunction of the PINK1 MTS is sufficient to initiate the downstream events in the PINK1–Parkin pathway, irrespective of ΔΨm.

## DISCUSSION

Sporadic Parkinson's disease and chemical-induced parkinsonism have been associated with mitochondrial defects. For example, toxic parkinsonism is caused by inhibitors of the mitochondrial electron transport chain complex I such as rotenone or 1-methyl-4-phenyl-1,2,3,6-tetrahydropyridine (MPTP) (Tanner et al., 2011). Moreover, reduction of respiratory chain complex I activity or mitochondrial DNA (mtDNA) mutations have been observed in some sporadic Parkinson's disease patients (Schapira, 2008; Schapira, 2010). Therefore, an aspect of Parkinson's disease might be caused by mitochondrial dysfunction attributable to a respiration complex I defect, mtDNA mutation or mitochondrial toxin; thus, the elimination of dysfunctional mitochondria is important to prevent Parkinson's disease. The MTS-possessing PINK1 kinase and Parkin ubiquitin ligase cooperate in mitochondrial quality control to protect against familial Parkinson's disease by removing damaged mitochondria (Okamoto, 2014; Trempe and Fon, 2013; Winklhofer, 2014). When ΔΨm is reduced, the driving force for matrix import decreases, resulting in the accumulation of activated PINK1 on depolarized mitochondria (Matsuda et al., 2010; Narendra et al., 2010; Okatsu et al., 2012b). However, in this process, the molecular basis for why PINK1 is not released into cytosol but is rather retained on depolarized mitochondria in the absence of the driving force for mitochondrial import has remained unresolved. Indeed, several MTS-containing matrix proteins such as Su9–GFP relocate to the cytosol and never localize on mitochondria following a decrease in ΔΨm (Fig. 2). Here, we addressed these questions by elucidating the molecular mechanism underlying the targeting of PINK1 to the OMM of depolarized mitochondria.

Residues 1–34 of the PINK1 N-terminus (referred to as N34) comprise a typical MTS that when fused to GFP (N34–GFP) resulted in the passage of the reporter protein through the IMM and into the mitochondrial matrix (Fig. 2). When MTS function was either inhibited by ΔΨm dissipation or disrupted by amino acid insertion, N34–GFP localized to the cytosol (Figs 2, 3). By contrast, disruption of MTS function in full-length PINK1 had no effect on mitochondrial localization (Fig. 4; supplementary material Fig. S4). These results suggest that PINK1 has an alternative mitochondrial localization mechanism that manifests when the N-terminal MTS becomes dysfunctional. Unexpectedly, siTom40, which is expected to prevent MTS import as well, inhibited PINK1 localization on both energized and depolarized mitochondria (Fig. 5). This result is seemingly inconsistent with the aforementioned hypothesis that the alternative mitochondrial localization mechanism is utilized when the N-terminal MTS is disrupted. However, we and other groups have reported that the TOM machinery interacts with PINK1 following a decrease in ΔΨm (Bertolin et al., 2013; Lazarou et al., 2012; Okatsu et al., 2013); thus, we surmise that the TOM machinery interacts with the alternative signal to facilitate OMM localization. Using several deletion mutants, we demonstrated that the N-terminal 90 amino acids of PINK1 are sufficient for OMM localization when the typical MTS is inhibited (Figs 4, 6), whereas the hydrophobic region (residues 35–90), that was defined *a priori* as a TMD, is dispensable for OMM localization (Fig. 7). Taken together with the database prediction (supplementary material Fig. S3), these data lead us to speculate that residues 70–94 of PINK1, which are upstream of the apparent TMD, potentially function as the alternative OMM localization signal.

Usually, almost all mitochondria generate a normal ΔΨm, which can be easily confirmed by ΔΨm-dependent dyes such as TMRE (Fig. 3D; Fig. 4B). Presumably, very small populations of mitochondria become depolarized, but are disengaged from the fusion-fission cycle and are ultimately eliminated by mitochondria-dedicated autophagy (mitophagy) (Twig et al., 2008). We demonstrated in this study that PINK1 OMM localization, autophosphorylation and Parkin recruitment are autonomously induced once MTS-dependent PINK1 matrix import is inhibited (Figs 4, 8; supplementary material Fig. S4); other special preconditions for PINK1 activation are not required. This mechanism seems appropriate because dysfunctional mitochondria that occasionally emerge in large populations of intact mitochondria should be removed selectively. We also showed that ΔN34 PINK1, N-FLAG–PINK1, and N-term 5D PINK1 mutants were converted to the activated form even on energized mitochondria. From a different viewpoint, these mutants behave as constitutively active PINK1 mutants. It has been reported that constitutively active forms of kinases such as MAPKK have been effectively used to analyze their physiological function (Cowley et al., 1994; Mansour et al., 1994). The discovery of a constitutively active form of PINK1 would also enable us to study PINK1 function separately from mitochondrial depolarization, and would be a good tool to study both PINK1 and Parkin.

In summary, our proposed molecular mechanism underlying PINK1 activation on depolarized mitochondria is illustrated in Fig. 8G. PINK1 possesses three domains that are important for submitochondrial localization – (1) the N-terminal MTS for matrix import, (2) the TMD for retention in IMM that subsequently provides a degradation signal and (3) the outer mitochondrial localization signal (OMS) elucidated in this study that is located between the MTS and TMD. PINK1 is usually degraded in energized mitochondria in an MTS- and TMD-dependent manner (Fig. 8G, left panel). However, dysfunction of the MTS targets PINK1 to the OMM by a usually latent OMS. As a consequence, PINK1 is activated and transduces the signal to Parkin (Fig. 8G, right panel). Interestingly, the OMS does not depend on ΔΨm (Fig. 6), but instead is contingent on the TOM machinery (Fig. 5). This feature is reminiscent of the N-terminal signal-anchored proteins that localize to the OMM in a ΔΨm-independent and Tom40-dependent manner, and which have the bulk of the polypeptide exposed to the cytosol (Ahting et al., 2005). PINK1 thus might be considered to be a unique N-terminal signal-anchored protein containing an extra MTS and an IMM-arrival-dependent degradation signal (TMD).

## MATERIALS AND METHODS

### Plasmids, siRNA and antibodies

The plasmids used in this study are summarized in supplementary material Table S1. The plasmid for weak PINK1 expression, pCMVTNT(d1), was generated by deleting the upstream 620 bp of the CMV promoter in pCMVTNT (Promega), as described previously (Okatsu et al., 2012b). For siRNA analysis, siGENOME siRNA SMART pool (M-012732-00-0005, Thermo Fisher Scientific) and siGENOME non-targeting siRNA pool (D-001206-13-20, Thermo Fisher Scientific) were used for the knockdown of Tom40 and as a control, respectively. The following primary antibodies were used: anti-Parkin (clone PRK8, Sigma, 1∶2000), anti-PINK1 (BC100-494, Novus, 1∶1000), anti-GFP (ab6556, Abcam, 1∶2000), anti-Tom20 (FL-145, Santa Cruz Biotechnology, 1∶250 for immunoblotting or 1∶2000 for immunostaining), anti-Tom22 (clone 1C9-2, Sigma, 1∶1000), anti-OPA1 (clone 18, BD Biosciences, 1∶500), anti-AIF (clone E-1, Santa Cruz, 1∶500), anti-Hsp60 (clone N-20, Santa Cruz Biotechnology, 1∶500), anti-cytochrome c (6H2.B4, BD Biosciences, 1∶150), anti-FoF1 ATPase (provided by Dr Takashi Ueno, Juntendo University, Tokyo, Japan; 1∶300) and anti-Tom40 (provided by Dr Katsuyoshi Mihara, Kyushu University, Fukuoka, Japan; 1∶1000). We also used the following secondary antibodies: anti-mouse-IgG, anti-rabbit-IgG or anti-goat-IgG antibody conjugated to alkaline phosphatase (Santa Cruz, 1∶5000) for immunoblotting, and anti-mouse-IgG or anti-rabbit-IgG antibody conjugated to Alexa Fluor 488, 568 or 647 (Life Technologies, 1∶2000) for immunostaining.

### Cells and DNA or siRNA transfection

HeLa cells were cultured at 37°C with 5% CO_2_ in Dulbecco's modified Eagle medium (DMEM, Sigma) containing 10% fetal bovine serum (Equitech-BIO. Inc.), 1× penicillin-streptomycin-glutamine (Life Technologies), 1× non-essential amino acids (Life Technologies) and 1× sodium pyruvate (Life Technologies). HeLa cells were transfected with the plasmids used in this study by using Fugene 6 (Roche or Promega). For expression of wild-type or mutant PINK1 in Tom40-knockdown cells, Tom40 and control siRNAs were introduced into HeLa cells using RNAiMAX reagent (Life Technologies), cells were incubated for 72 h and then PINK1 plasmids were introduced into these cells using Fugene 6, followed by incubation for a further 24 h and then data collection.

### CCCP treatment

For mitochondrial depolarization, cells were treated with 10 µM CCCP for 1 h unless otherwise specified. To examine the ΔΨm-dependency of mitochondrial import of Su9–GFP or PINK1-N34–GFP, cells were pre-treated with CCCP for 2 h prior to transfection with Su9–GFP or PINK1-N34–GFP and further incubated in the presence of CCCP. Following an 18-h incubation, the subcellular localizations of these GFP-fused proteins were observed.

### Immunofluorescence, TMRE staining and MitoTracker staining

For immunofluorescence experiments, cells were fixed with 4% paraformaldehyde, permeabilized with 50 µg/ml digitonin or 1% Triton X-100, and then stained with the primary and secondary antibodies described above. Cells were imaged using a confocal laser-scanning microscope (LSM510, LSM710 and LSM780; Carl Zeiss, Inc.). To monitor the ΔΨm, cells were treated with 150 nM MitoTracker Orange CM-H2-TMRos (Life Technologies) or 50 nM TMRE (Sigma) for 30 min. Cells were washed two to three times and fixed as above for MitoTracker staining or subjected to live-cell imaging for TMRE staining. Image contrast and brightness were adjusted in Photoshop (Adobe).

### Mitochondrial-enriched fractionation and proteinase K protection assay

HeLa cells transfected with PINK1-N34–GFP were suspended in fractionation buffer [250 mM sucrose, 20 mM HEPES-NaOH pH 8.1, protease- and phosphatase-inhibitor cocktail (Roche)] and disrupted by 30 passages through a 25-gauge needle using a 1-ml syringe. Debris was removed by centrifugation at 1000 ***g*** for 7 min, and the supernatant was subjected to centrifugation at 10,000 ***g*** for 10 min at 4°C to precipitate the mitochondria-rich fraction. The mitochondria-rich fractions were incubated with 200 µg/µl Pro K (Wako chemicals) on ice for 30 min. The reaction was stopped with 1 mM phenylmethylsulfonyl fluoride (PMSF) before boiling for electrophoresis.

### Phos-tag SDS-PAGE and immunoblotting

To detect phosphorylated proteins, SDS-PAGE with or without 50 µM Phos-tag acrylamide (Wako chemicals) and 100 µM MnCl_2_ were used. After electrophoresis, Phos-tag acrylamide gels were washed using transfer buffer with 0.01% SDS and 1 mM EDTA for 10 min with gentle shaking and then replaced with transfer buffer with 0.01% SDS without EDTA for 10 min according to the manufacturer's protocol. Proteins were transferred to PVDF membranes and detected by the indicated antibodies using standard immunoblotting procedures.

## Supplementary Material

Supplementary Material
