# Supplementary material for: Unconventional PINK1 localization to the outer membrane of depolarized mitochondria drives Parkin recruitment
Source: J Cell Sci. 2015 Mar 1;128(5):964–78. doi: 10.1242/jcs.161000 (PMC4342580; doi:10.1242/jcs.161000)
Supplement: Supplementary Material [file supp_128_5_964__index.html]

Unconventional PINK1 localization to the outer membrane of depolarized mitochondria drives Parkin recruitment — Supplementary Material 

# Unconventional PINK1 localization to the outer membrane of depolarized mitochondria drives Parkin recruitment

## JCS161000 Supplementary Material

**Files in this Data Supplement:**

- **Supplementary Material**
