# Supplementary material for: Unconventional PINK1 localization to the outer membrane of depolarized mitochondria drives Parkin recruitment
Source: J Cell Sci. 2015 Mar 1;128(5):964–78. doi: 10.1242/jcs.161000 (PMC4342580; doi:10.1242/jcs.161000)
Supplement: Supplementary Material [file supp_128.5.964_JCS161000.pdf]

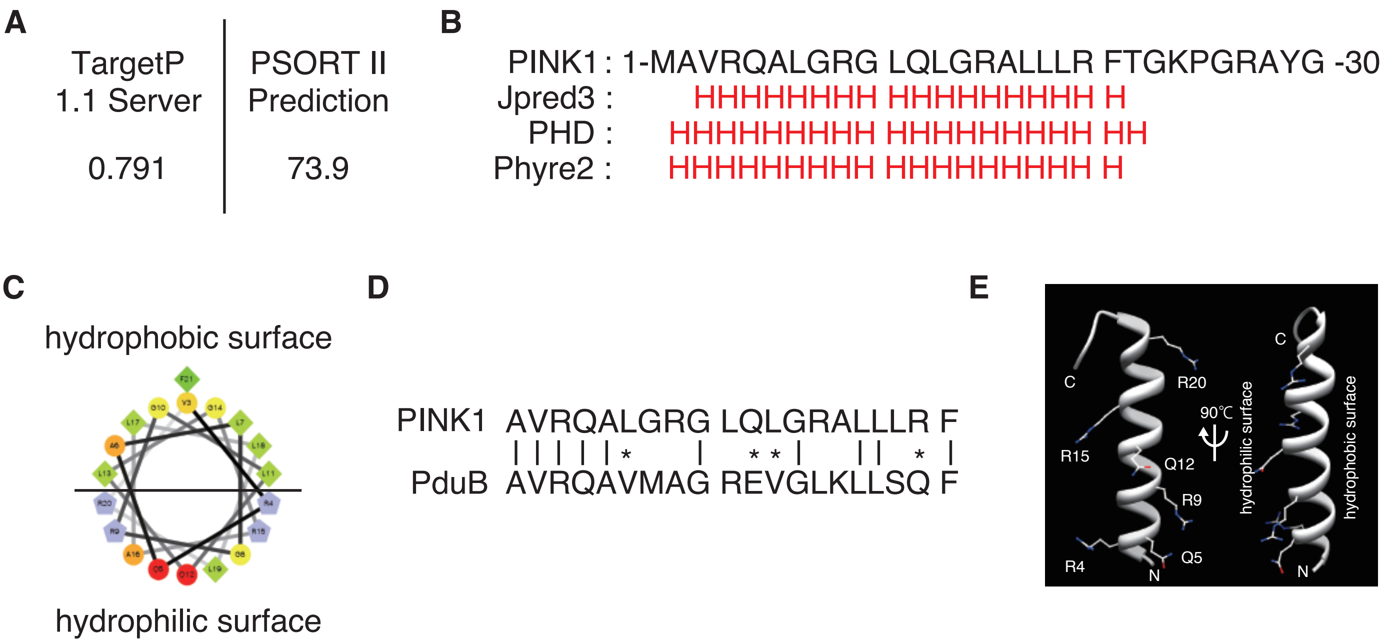

Fig. S1. The most N-terminal region of PINK1 has characteristics of a MTS.

A. TargetP 1.1 and PSORT II mitochondrial localization prediction scores for PINK1. **B**. Jpred3, PDH, and Phyre2 predict an alpha helical segment in the PINK1 N-terminus. The red “H” indicates predicted helicity. **C**. Helical wheel simulated from the predicted PINK1 helical region indicates an amphiphilic alpha helix. **D**. Sequence comparison between PINK1 and BduB. The bars and asterisks indicate identical and similar amino acids, respectively. **E.** Modeling of the predicted PINK1 amphiphilic alpha helix based on PduB using Phyre2. Hydrophilic amino acids are shown as side chains. N and C indicate N-terminus and C-terminus, respectively.

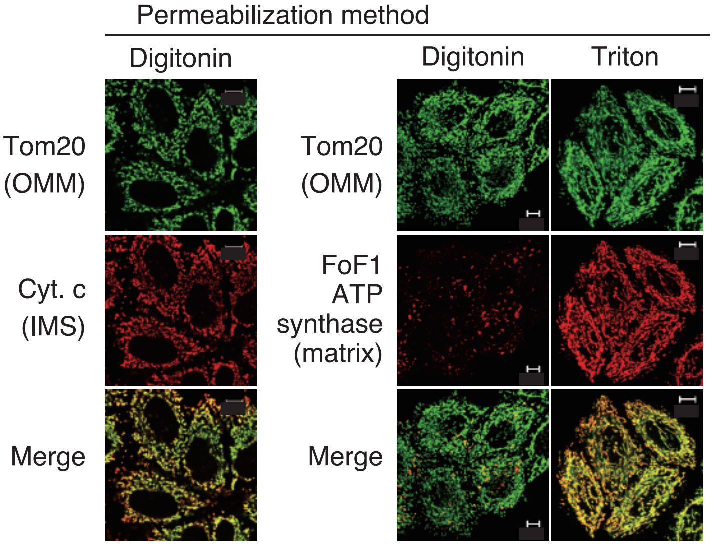

Fig. S2. Triton X-100 permeabilization is required to detect mitochondrial matrix proteins by immunocytochemistry, whereas digitonin permeabilization cannot detect the signal.

Immunocytochemistry using anti-Tom20 (OMM protein), anti-Cytochrome c (Cyt c) (IMS protein), and anti-FoF1 ATPase (matrix-facing protein) antibodies following permeabilization with 50 μg/mL digitonin or 1% Triton X-100 were shown. Scale bars: 10 μm.

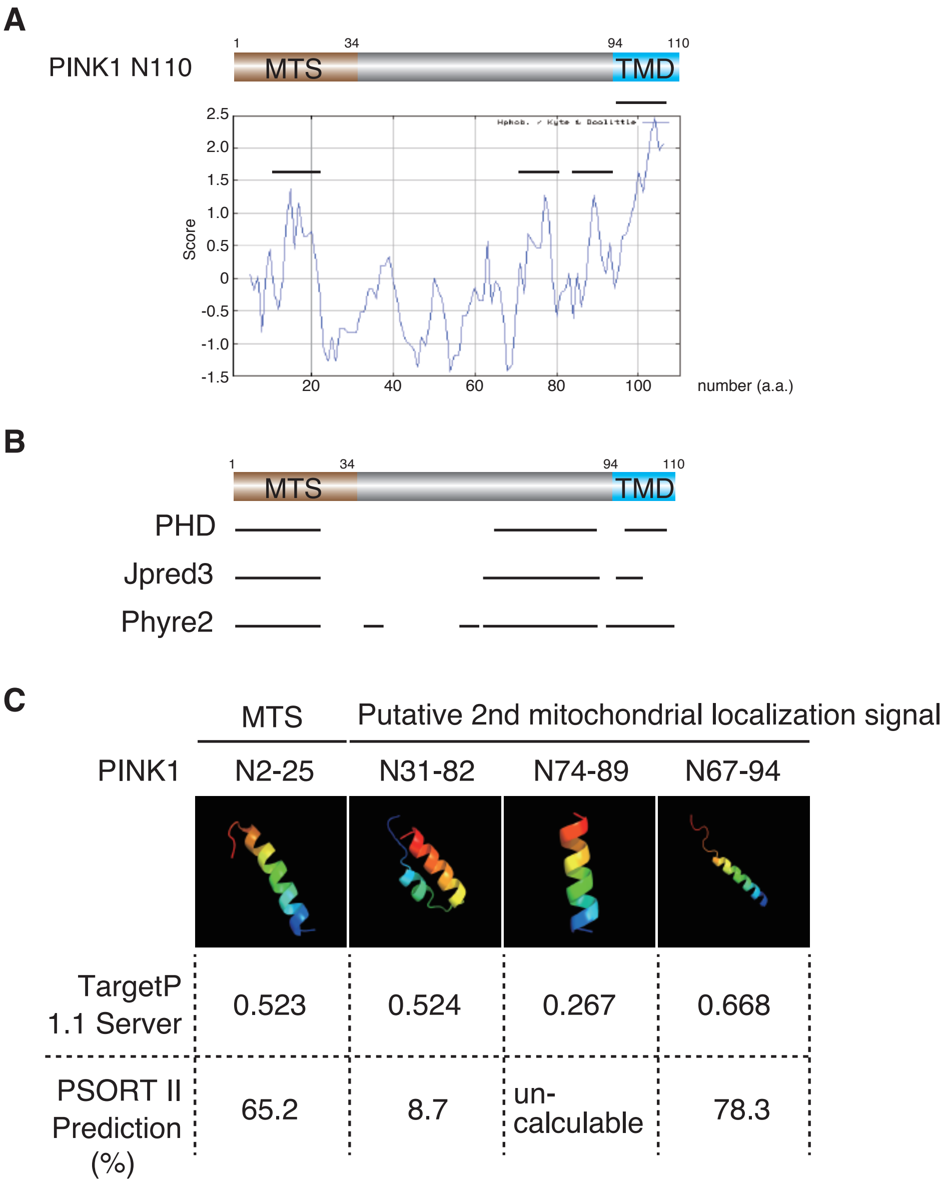

Fig. S3. Possible mitochondrial localization signal in residues 70 – 90 of the PINK1 N terminus.

A. Hydropathy profile of residues 1-110 of the PINK1 N terminus. Bars indicate hydrophobic regions.

B. Jpred3 (http://www.compbio.dundee.ac.uk/www-jpred/),

PDH (http://npsa-pbil.ibcp.fr/cgi-bin/npsa_automat.pl?page=/NPSA/npsa_phd.html), and

Phyre2 (http://www.sbg.bio.ic.ac.uk/~phyre2/html/) suggest an alpha helix around residues 70 - 95. Bars indicate the predicted helical structural region. **C**. The expected values for mitochondrial localization of the indicated region of PINK1 calculated by TargetP 1.1 (http://www.cbs.dtu.dk/services/TargetP/) and PSORT II (http://psort.hgc.jp/form2.html). Predicted helical structures are visualized by Phyre2.

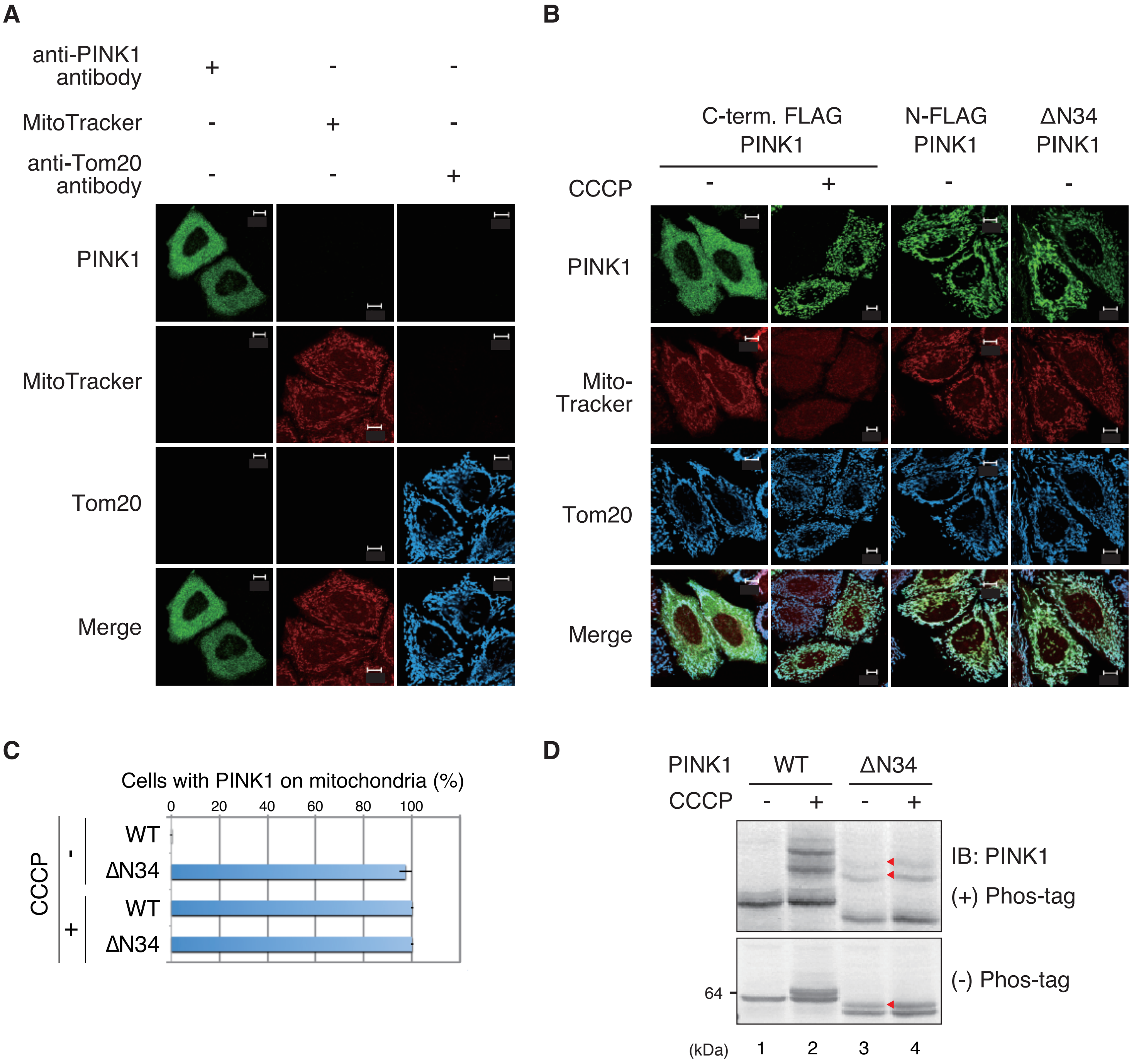

Fig. S4. PINK1 lacking the N-terminal MTS localizes on energized mitochondria and is phosphorylated.

A. HeLa cells expressing PINK1 were stained individually with an anti-PINK1 antibody, the fixable ΔΨm-dependent dye MitoTracker Orange CM-H2TMRos, or an anti-Tom20 antibody. Data confirmed no channel cross talk between the signals. **B**. HeLa cells expressing C-terminal tagged, N-terminal tagged, and N-terminal deleted ΔN34 PINK1 were stained with MitoTracker Orange CM-H2TMRos, then immunostained with anti-PINK1 and anti-Tom20 antibodies. Scale bars indicate 10 μm in (A) and (B). **C**. The number of cells with mitochondria-localized WT or ΔN34 PINK1 was counted. Error bars represent the mean ± SD values of at least three experiments in 100 cells. **D**. The cells expressing ΔN34 PINK1 were subjected to SDS-PAGE ± Phos-tag. The red arrowheads indicate phosphorylated PINK1 under steady state conditions.

Supplementary Table. List of plasmids used.

Vector	description	source
pCMV14-PINK1-3Flag	For transient expression of WT PINK1-3Flag	Shiba, K. *et al*. *Biochem Biophys Res Commun* 383, 331-335 (2009)
pcDNA3.1 non-tagged PINK1	For expression of non-tagged WT PINK1	Takatori, S. *et al*. *Neurosci Letters* 430, 13-17 (2008)
pCMV3.1-Flag-PINK1 WT	For expression of Flag-WT PINK1	Matsuda, N. *et al*. *J*. *Cell Biol*. 189, 211-221 (2010)
pCMVTNT -PINK1-3HA	For expression of WT PINK-3HA	Okatsu, K. *et al*. *Nat Commun* 3, 1016 (2012)
pCMVTNT(d1)-PINK1-3HA	For weak expression of WT PINK1-3HA	Okatsu, K. *et al*. *Nat Commun* 3, 1016 (2012)
pEGFP-C1-GFP-Parkin	For weak expression of GFP-WT Parkin	Matsuda, N. *et al*. *J*. *Cell Biol*. 189, 211-221 (2010)
pEGFP-N1	For expression of GFP	Clontech
pEGFP-N1-Su9	For expression of Su9-GFP	Ishihara, N. *et al*. *Biochem Biophys Res Commun* 301, 891-98 (2003)
pEGFP-N1-PINK1 N34	For expression of PINK1 N34-GFP	Takatori, S. *et al*. *Neurosci Letters* 430, 13-17 (2008)
pEGFP-N1-3Ala-PINK1 N34	For expression of PINK1 N34-GFP inserted 3Ala	This study
pEGFP-N1-5Ala-PINK1 N34	For expression of PINK1 N34-GFP inserted 5Ala	This study
pEGFP-N1-3Asp-PINK1 N34	For expression of PINK1 N34-GFP inserted 3Asp	This study
pEGFP-N1-5Asp-PINK1 N34	For expression of PINK1 N34-GFP inserted 5Asp	This study
pEGFP-N1-PINK1	For expression of WT PINK1-GFP	Okatsu, K. *et al*. *Nat Commun* 3, 1016 (2012)
pCMV3.1-non-tag PINK1Δ 91-111 a.a.	For expression of PINK1 ΔTMD	This study
pEGFP-N1-3Asp-PINK1	For expression of PINK1-GFP inserted 5Asp	This study
pEGFP-N1-5Asp-PINK1	For expression of PINK1-GFP inserted 5Asp	This study
pCMV14-ΔN35-3Flag	For expression of PINK1 deleted N35 a.a.	This study
pCMVTNT(d1)-ΔN35-3HA	For weak expression of PINK1 deleted N35 a.a.	This study

*1. WT means wild type. *2. a.a. means amino acid
